# The Parkinson’s Real-World Impact Assessment (PRISM) Study: A European Survey of the Burden of Parkinson’s Disease in Patients and their Carers

**DOI:** 10.3233/JPD-212611

**Published:** 2021-08-02

**Authors:** Eduardo Tolosa, Georg Ebersbach, Joaquim J. Ferreira, Olivier Rascol, Angelo Antonini, Thomas Foltynie, Rachel Gibson, Diogo Magalhaes, J. Francisco Rocha, Andrew Lees

**Affiliations:** a Hospital Clínic de Barcelona, University of Barcelona, Barcelona, Spain; b Movement Disorders Clinic, Beelitz-Heilstätten, Germany; c Laboratory of Clinical Pharmacology and Therapeutics, Faculdade de Medicina, Universidade de Lisboa, Lisbon, Portugal; d Toulouse Parkinson Expert Center, Departments of Neurosciences and Clinical Pharmacology, Centre d’Investigation Clinique de Toulouse CIC1436, NS-Park/FCRIN Network, and NeuroToul COEN Center, University Hospital of Toulouse, INSERM, University of Toulouse, Toulouse, France; e Parkinson Disease and Movement Disorder Unit, Department of Neurosciences, University of Padova, Padova, Italy; f Department of Clinical and Movement Neurosciences, National Hospital for Neurology and Neurosurgery, London, United Kingdom; g The Cure Parkinson’s Trust, London, UK; h BIAL –Portela & C^a^ S.A., Coronado, Portugal

**Keywords:** Caregivers, catechol o-methyltransferase inhibitors, comorbidity, dopamine agonists, Europe, levodopa, observational study, Parkinson’s disease, quality of life, surveys and questionnaires

## Abstract

**Background:**

A greater understanding of the everyday experiences of people with Parkinson’s disease (PD) and their carers may help improve clinical practice.

**Objective:**

The Parkinson’s Real-world Impact assesSMent (PRISM) study evaluated medication use, health-related quality of life (HRQoL) and the use of healthcare resources by people with PD and their carers.

**Methods:**

PRISM is an observational cross-sectional study, in which people with PD and their carers completed an online survey using structured questionnaires, including the Parkinson’s Disease Quality of Life Questionnaire (PDQ-39), Non-Motor Symptoms Questionnaire (NMSQuest) and Zarit Burden Interview (ZBI).

**Results:**

Data were collected from 861 people with PD (mean age, 65.0 years; mean disease duration, 7.7 years) and 256 carers from six European countries. People with PD reported a large number of different co-morbidities, non-motor symptoms (mean NMSQuest score, 12.8), and impaired HRQoL (median PDQ-39 summary score, 29.1). Forty-five percent of people with PD reported at least one impulse control behaviour. Treatment patterns varied considerably between different European countries. Levodopa was taken in the last 12 months by 85.9% of participants, and as monotherapy by 21.8%. Carers, who were mostly female (64.8%) and the partner/spouse of the person with PD (82.1%), reported mild to moderate burden (mean ZBI total score, 26.6).

**Conclusions:**

The PRISM study sheds light on the lives of people with PD and those who care for them, re-emphasising the many challenges they face in everyday life. The study also provides insights into the current treatment of PD in Europe.

## INTRODUCTION

Parkinson’s disease (PD) is the most common neurodegenerative movement disorder, with estimated prevalence and incidence rates in Europe of approximately 108—257/100,000 and 11—19/100,000 per year, respectively [1]. Data from the Global Burden of Disease Study have shown that the number of people with PD has more than doubled globally over the last 25 years to over 6 million, in part due to more people living for longer [2].

People with PD have to contend with increasing physical disability, a greater risk of dementia and depression, and treatment-related complications including dyskinesias and impulse control disorders [3–8], all of which can affect their health-related quality of life (HRQoL) [9]. The lives of carers are also affected, resulting in situational anxiety and depression and physical exhaustion, as well as financial hardship [9–13].

A greater understanding of the everyday experiences of people with PD may help to improve clinical practice and improve the quality of life of patients and those who care for them.

## MATERIALS AND METHODS

### Study design

The Parkinson’s Real-world Impact assesSMent (PRISM) study is a European, observational, cross-sectional survey designed by an international scientific committee in collaboration with The Cure Parkinson’s Trust (a United Kingdom-based research-driven charity). The data were collected using an online questionnaire, completed by people with PD and their carers (Supplementary Material 1). The questionnaire comprised two main sections: the first was completed from the perspective of the people with PD, either by themselves or with the help of their carers, and the second was completed by the primary carer. An initial pilot study was conducted in the United Kingdom (February–March 2019), following which the survey was modified to improve clarity and then translated for use in other European countries (France, Germany, Italy, Portugal and Spain). Data from the pilot study were included in the final analysis.

A process was undertaken in the United Kingdom to determine whether ethical approval was required for the study, using online tools provided by the NHS England Health Research Authority. This indicated that the study was research (‘*Is my study research?*’ http://www.hra-decisiontools.org.uk/research/) but did not require NHS Research Ethics Committee (REC) approval (‘*Do I need NHS REC approval?*’ http://www.hra-decisiontools.org.uk/ethics/). Participation in the study was voluntary for all respondents (including omitting individual survey questions that a respondent did not wish to answer). The survey was made available primarily via patient groups and at the discretion of selected healthcare centres (if ‘advertisement’ was required in order to extend reach in any of the participating countries, this was not in any way coercive, relying solely on leaflets in patient waiting rooms); and no identifying information about respondents was requested or held by researchers involved in the study. All participants were informed before entering the survey that all information would be treated confidentially and stored securely, as required by General Data Protection Regulation. Healthcare professionals had no direct role in recruitment.

### Study population

People with PD and their carers were recruited through the help of PD advocacy groups in each country, through email and social media campaigns; and leaflets made available at patient advocacy group events in Portugal, Spain and the United Kingdom, and in specialist PD clinics in Spain. Since participation in the online survey was voluntary, it was not possible to actively screen a patient sample that was representative of the whole PD population. However, recruitment efforts aimed to reach the maximum number of people with PD in each of the target countries. Advocacy groups (Supplementary Material 2) maintain online networks of people with PD, through regular newsletters, online forums and social media.

### Study assessments

It was advised that, if possible, most of the questions in the online questionnaire should be completed by people with PD and carers together. Sensitive questions (e.g., relating to sexual functioning) were optional and placed in a separate section at the end of the survey, where it was clearly indicated that these questions could be completed by the patient or carer alone.

#### Questionnaire for people with PD

Socio-demographic data and information on co-morbidities, pharmacological treatment, the use of healthcare resources and the impact of PD on employment, family relationships, sexual relationships and impulse control behaviour were obtained using structured questionnaires (Supplementary Material 1). HRQoL was assessed using the Parkinson’s Disease Quality of Life Questionnaire (PDQ-39; Oxford University Innovation Limited) [14] and non-motor symptoms were assessed using the Non-Motor Symptoms Questionnaire (NMSQuest; International Parkinson and Movement Disorder Society, Inc.) [15]. The PDQ-39 has been validated for use in all of the languages used in PRISM. The NMSQuest has been translated and validated for use in English, Spanish and German. Agreement for translation into French, Italian and Portuguese was obtained from the developer (translation conducted by UK Techtrans Ltd.). Impulsivity assessment was based on the Questionnaire for Impulsive-Compulsive Disorder in Parkinson’s Disease (QUIP) [16], where patients were asked whether they, or others close to them, thought that they had problems related to gambling, hypersexuality, buying too much, eating too much, taking too much PD medication, or spending too much time on hobbies (‘hobbyism’). Questions relating to sexual relationships were taken from the Medical Outcomes Study Sexual Functioning Scale [17]. Questions relating to demographics, comorbidities and employment status were collected without specific tools/questionnaires.

#### Carer questionnaire

Socio-demographic data, including information on the carer’s relationship to the person with PD, the number of hours spent caring for the person with PD, the use of social network support to help with care, and the impact of PD on the carer’s relationship with the patient, were obtained by a structured interview (Supplementary Material 1). Carer burden was assessed using the Zarit Burden Interview (ZBI; Mapi Research Trust) [18, 19]. The ZBI comprises 22 questions about the impact of the patient’s disabilities on the carer’s life. Answers are scored 0 for ‘never’, 1 for ‘rarely’, 2 for ‘sometimes’, 3 for ‘quite frequently’ and 4 for ‘nearly always’, with the total scores ranging from 0–88 (0–20, little or no burden; 21–40, mild to moderate burden; 41–60, moderate to severe burden; 61–88, severe burden [20]). The ZBI has been validated for use in all of the languages used in PRISM.

### Statistical methods

A target of 100 responses was set for each country. While the study was not powered to demonstrate statistical differences, representation of population sub-groups (patient age, nature of therapeutic intervention) was attempted. No formal statistical analyses were performed. Continuous variables were summarised using descriptive statistics, and categorical variables were summarised using frequency counts and percentages.

## RESULTS

### Study population

Between 11 April 2019 and 31 July 2019, data were collected from 861 people with PD (of whom 599 provided complete responses and 262 provided partial responses) and from 256 carers from six European countries (France, Germany, Italy, Portugal, Spain and the United Kingdom). ‘Complete response’ was defined as reaching the end of the non-optional questions (all questions up to and including Q84; see Supplementary Material 1) before submitting the survey responses. Of the 599 respondents who reached the end of the non-optional questions, a small proportion did not reply to all previous questions (PDQ-39, *n* = 1; Q10, *n* = 11; Q83, *n* = 11). ‘Partial response’ was defined as failure to meet the criterion for complete response.

### Characteristics of people with PD

The mean age of the studied population was 65.0 years (ranging from 62.2 years in Germany to 68.8 years in France) and 50.5% were male (Table 1). The majority of participants (85.9%) were aged between 50 and 79 years. The mean age at diagnosis was 57.7 years (ranging from 54.3 years in Germany to 59.8 years in France) and the mean disease duration was 7.7 years (ranging from 6.2 years in the United Kingdom to 9.5 years in France). Most of the participants lived in urban locations, since 80.8% travelled < 30 miles/50 km to see a specialist. The population was well educated, with 34.0% having a university degree or post-graduate degree, and < 20% having primary or secondary non-advanced school/vocational training as their highest education level.

**Table 1:** Characteristics of people with PD in the PRISM cohort, by country

Characteristic	Total	France	Germany	Italy	Portugal	Spain	United Kingdom
Number of respondents
N	861	63	92	264	80	149	213
Complete response, *n* (%)	599 (69.6)	39 (61.9)	65 (70.7)	172 (65.2)	53 (66.3)	100 (67.1)	170 (79.8)
Partial response, *n* (%)	262 (30.4)	24 (38.1)	27 (29.3)	92 (34.8)	27 (33.8)	49 (32.9)	43 (20.2)
Gender
N	858	62	92	264	80	149	211
Male, *n* (%)	433 (50.5)	33 (53.2)	46 (50.0)	135 (51.1)	44 (55.0)	78 (52.4)	97 (46.0)
Female, *n* (%)	418 (48.7)	29 (46.8)	45 (48.9)	126 (47.7)	36 (45.0)	70 (47.0)	112 (53.1)
Other, *n* (%)	4 (0.5)	0	0	2 (0.8)	0	1 (0.7)	1 (0.5)
Prefer not to say, *n* (%)	3 (0.4)	0	1 (1.1)	1 (0.4)	0	0	1 (0.5)
Age, y
N	855	62	92	262	80	148	211
Mean (SD)	65.0 (10.2)	68.8 (9.1)	62.2 (8.7)	65.9 (10.4)	66.2 (11.5)	62.6 (11.4)	65.4 (8.9)
Median (IQR)	65 (58–72)	70 (64–74)	61 (54–69)	66 (59–73)	66 (61–72)	62 (54–71)	66 (58–72)
Age group
N	856	62	92	262	80	149	211
< 40 y, *n* (%)	10 (1.2)	1 (1.6)	0	1 (0.4)	4 (5.0)	4 (2.7)	0
40–49 y, *n* (%)	44 (5.1)	0	4 (4.4)	14 (5.3)	4 (5.0)	13 (8.7)	9 (4.3)
50–59 y, *n* (%)	206 (24.1)	6 (9.7)	36 (39.1)	59 (22.5)	9 (11.3)	45 (30.2)	51 (24.2)
60–69 y, *n* (%)	295 (34.5)	23 (37.1)	31 (33.7)	87 (33.2)	33 (41.3)	45 (30.2)	76 (36.0)
70–79 y, *n* (%)	234 (27.3)	24 (38.7)	20 (21.7)	77 (29.4)	20 (25.0)	28 (18.8)	65 (30.8)
80–89 y, *n* (%)	64 (7.5)	8 (12.9)	1 (1.1)	22 (8.4)	10 (12.5)	13 (8.7)	10 (4.7)
≥90 y, *n* (%)	3 (0.4)	0	0	2 (0.8)	0	1 (0.7)	0
Age at diagnosis, y
N	827	51	90	261	79	137	209
Mean (SD)	57.7 (11.3)	59.8 (12.5)	54.3 (10.2)	57.6 (11.3)	58.2 (11.9)	56.5 (12.9)	59.2 (9.7)
Median (IQR)	58 (49–65)	60 (53–66)	54 (47–64)	57 (49–67)	58 (52–67)	55 (48–63)	59 (53–66)
Disease duration, y
N	813	48	90	258	77	131	209
Mean (SD)	7.7 (6.3)	9.5 (6.8)	7.7 (6.9)	8.4 (6.5)	8.8 (6.8)	7.6 (6.5)	6.2 (5.1)
Median (IQR)	6 (3–11)	9 (4–13)	6 (3–10)	7 (3–12)	7 (4–12)	6 (3–10)	5 (3–9)
Distance to travel to see a specialist
N	858	62	92	264	80	149	211
< 30 miles/50 km, *n* (%)	693 (80.8)	43 (69.4)	78 (84.8)	202 (76.5)	64 (80.0)	125 (83.9)	181 (85.8)
30–60 miles/50–100 km, *n* (%)	88 (10.3)	16 (25.8)	5 (5.4)	26 (10.0)	7 (8.8)	14 (9.4)	20 (9.5)
> 60 miles/100 km, *n* (%)	65 (7.6)	3 (4.8)	4 (4.4)	33 (12.5)	9 (11.3)	10 (6.7)	6 (2.8)
Unknown, *n* (%)	12 (1.4)	0	5 (5.4)	3 (1.1)	0	0	4 (1.9)
Highest education level
N	858	62	92	264	80	149	211
Primary or secondary school/vocational, *n* (%)	170 (19.8)	3 (4.8)	19 (20.7)	63 (23.9)	23 (28.8)	39 (26.2)	23 (10.9)
Secondary school advanced/vocational, *n* (%)	158 (18.4)	17 (27.4)	34 (37.0)	75 (28.4)	4 (5.0)	7 (4.7)	21 (10.0)
Further education or training college, *n* (%)	171 (19.9)	20 (32.3)	10 (10.9)	47 (17.8)	18 (22.5)	27 (18.1)	49 (23.2)
Some university, *n* (%)	51 (5.9)	6 (9.7)	0	5 (1.9)	8 (10.0)	19 (12.8)	13 (6.2)
Completed university degree, *n* (%)	190 (22.1)	8 (12.9)	22 (23.9)	58 (22.0)	17 (21.3)	38 (25.5)	47 (22.3)
Post-graduate degree, *n* (%)	102 (11.9)	8 (12.9)	4 (4.4)	16 (6.1)	7 (8.8)	17 (11.4)	50 (23.7)
Prefer not to say, *n* (%)	16 (1.9)	0	3 (3.3)	0	3 (3.8)	2 (1.3)	8 (3.8)
Most frequently reported co-morbidities^a^
N	859	63	92	264	80	149	211
High blood pressure	217 (25.3)	10 (15.9)	30 (32.6)	69 (26.1)	16 (20.0)	41 (27.5)	51 (24.2)
Depression	188 (21.9)	6 (9.5)	18 (19.6)	67 (25.4)	21 (26.3)	32 (22.8)	42 (19.9)
Anxiety	136 (15.8)	9 (14.3)	1 (1.1)	46 (17.4)	21 (26.3)	26 (17.5)	33 (15.6)
Rheumatic diseases	91 (10.6)	8 (12.7)	3 (3.3)	32 (12.1)	8 (10.0)	21 (14.1)	19 (9.0)
Heart issues	73 (8.5)	6 (9.5)	6 (6.5)	27 (10.2)	7 (8.8)	15 (10.1)	12 (5.7)
Diabetes	55 (6.4)	7 (11.1)	11 (12.0)	14 (5.3)	2 (2.5)	7 (4.7)	14 (6.6)
Asthma	49 (5.7)	2 (3.2)	9 (9.8)	10 (3.8)	2 (2.5)	4 (2.7)	22 (10.4)
Gastric ulcer	47 (5.5)	1 (1.6)	5 (5.4)	25 (9.5)	5 (6.3)	6 (4.0)	5 (2.4)
Cancer	43 (5.0)	6 (9.5)	1	17 (6.4)	0	6 (4.0)	13 (6.2)
Dementia	36 (4.2)	0	2 (2.2)	14 (5.3)	6 (7.5)	4 (2.7)	10 (4.7)
Peripheral vascular disease	31 (3.6)	3 (4.8)	8 (8.7)	11 (4.2)	3 (3.8)	4 (2.7)	2 (0.9)
Kidney disease	16 (1.9)	0	2 (2.2)	5 (1.9)	2 (2.5)	7 (4.7)	0

^a^≥4% of participants in any country. IQR, interquartile range; PD, Parkinson’s disease; SD, standard deviation.

A range of co-morbidities were reported with the most frequent (≥10% of participants) being hypertension (25.3%), depression (21.9%), anxiety (15.8%) and rheumatological conditions (10.6%) (Table 1).

### Use of anti-PD medication

**Fig. 1:**
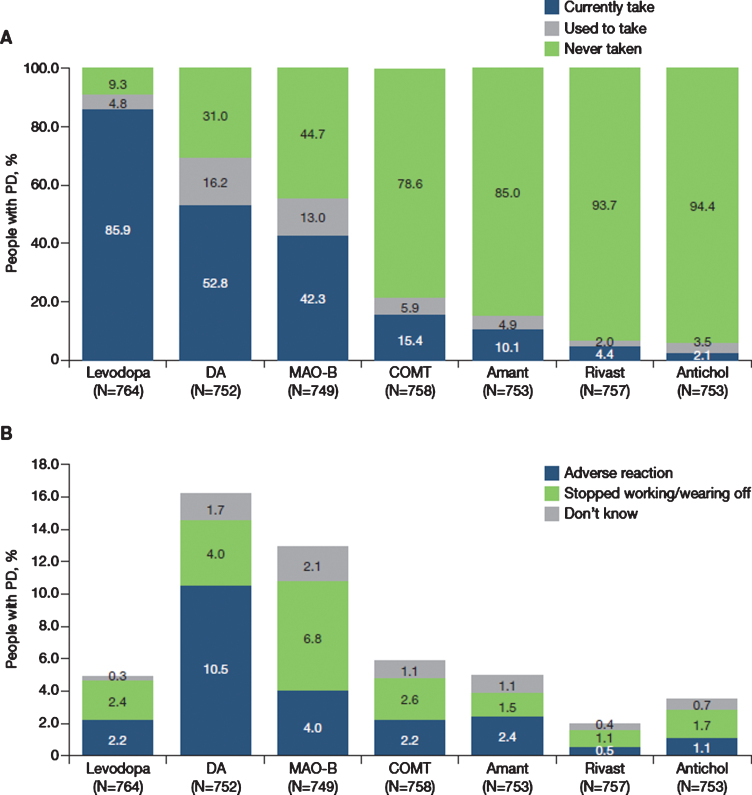
A) Current (last 12 months) and previous use of anti-PD medications by therapeutic class and B) Reasons for stopping use of therapeutic classes. N excludes missing values, “prefer not to say” and “other”. Antichol, anticholinergics; Amant, amantadine; COMT, catechol-O-methyltransferase inhibitor; DA, dopamine agonist; Levodopa, levodopa-containing therapy; MAO-B, monoamine oxidase-B inhibitor; Rivast, rivastigmine; PD, Parkinson’s disease.

**Fig. 2:**
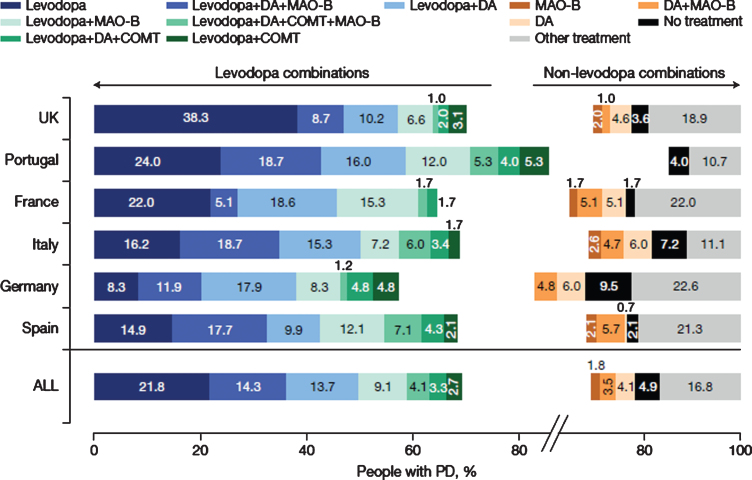
Current (last 12 months) use of therapeutic combinations (12 most common) for total PRISM population and by country. N = 790. N excludes missing values, “prefer not to say” and “other. COMT, catechol-O-methyltransferase inhibitor; DA, dopamine agonist; L-dopa, levodopa-containing therapy; MAO-B, monoamine oxidase-b inhibitor; PD, Parkinson’s disease.

Levodopa had been taken in the last year by 85.9% of participants (Fig. 1A) and was the first prescribed anti-PD medication in 67.4%, ranging from 58.2% in France to 87.5% in Portugal. Levodopa was taken as monotherapy by 21.8% of the overall population, ranging from 8.3% in Germany to 38.3% in the United Kingdom (Fig. 2). The use of levodopa increased with age: levodopa was used by 65.8% of people with PD aged 40–49 years, 78.7% of those aged 50–59 years, 88.1% of those aged 60–69 years, 88.4% of those aged 70–79 years and 89.8% of those aged 80–89 years. Dopamine agonists and MAO-B inhibitors were taken as monotherapy by 4.1% and 1.8% of participants in the overall population, respectively.

Dopamine agonists, MAO-B inhibitors and COMT inhibitors were currently taken (last 12 months) by 52.8%, 42.3% and 15.4% of people with PD, respectively (Fig. 1A). Of all the anti-PD classes, dopamine agonists were the anti-PD medication that was most commonly discontinued (16%), followed by MAO-B inhibitors (13%) and COMT inhibitors (6%) (Fig. 1A). The commonest reason for stopping treatment with a dopamine agonist was an adverse reaction (10.5%), whereas the most common reason for stopping treatment with both MAO-B and COMT inhibitors was ‘stopped working or re-emergence of wearing off’ effects (MAO-B inhibitors, 6.8%; COMT inhibitors, 2.6%) (Fig. 1B).

The most common combinations of PD medications in the overall population were levodopa +dopamine agonist + MAO-B inhibitor (14.3%), followed by levodopa + dopamine agonist (13.7%) and levodopa + MAO-B inhibitor (9.1%). However, there were notable differences between countries (Fig. 2). For example, levodopa + dopamine agonist + MAO-B inhibitor was the commonest combination in Italy (18.7%), Portugal (18.7%) and Spain (17.7%), whereas levodopa + dopamine agonist were most often used in France (18.6%), Germany (17.9%) and the United Kingdom (10.2%). The number of participants receiving no anti-Parkinsonian drug treatment ranged from 1.7% to 9.5% 

### Impact of PD on quality of life

**Fig. 3:**
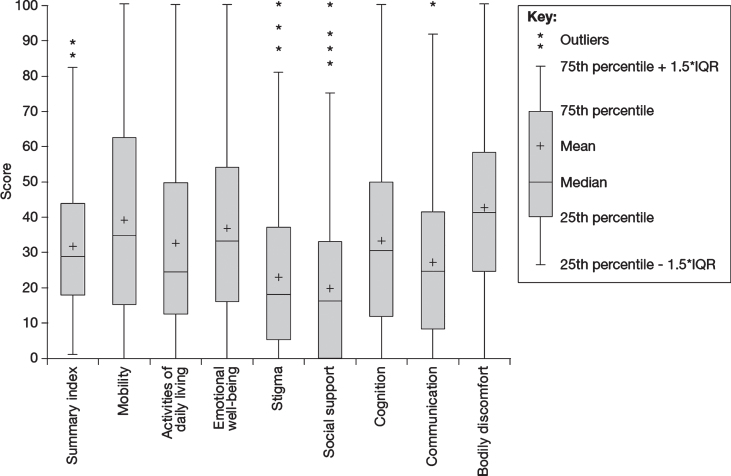
HRQoL in people with PD as measured using the PDQ-39 (*N* = 859). HRQoL, health-related quality of life; IQR, interquartile range; PD, Parkinson’s disease; PDQ-39, Parkinson’s Disease Questionnaire-39.

HRQoL and factors that have an impact on the HRQoL of people with PD were measured using several instruments, including the PDQ-39, the NMSQuest, and a structured interview to investigate employment, engagement in daily activities, impulse control, sexual functioning and relationships. Results of the PDQ-39 demonstrated that people with PD had impaired HRQoL (Fig. 3; Table 2). The median PDQ-39 summary score was 29.1 (interquartile range [IQR], 18.0–43.9), with the highest domain scores (i.e., worst HRQoL) occurring in bodily discomfort (median, 41.7; IQR, 25.0–58.3) and mobility (median, 35.0; IQR, 15.0–62.5). PDQ-39 scores showed worse HRQoL in those diagnosed before age 50 years across all domains except cognition (median summary score, 34.8 vs. 31.0) (full data not shown). PDQ-39 scores were also higher across all domains in people with PD diagnosed with anxiety and/or depression than in those not diagnosed with either condition (median summary score, 46.2 vs. 28.6) (full data not shown).

**Table 2:** HRQoL (measured using the PDQ-39), non-motor symptoms (measured using the NMSQuest), employment status and retirement status in people with PD

Characteristic	Statistic
PDQ-39 summary score
N	859
Median (IQR)	29.1 (18.0–43.9)
NMSQuest score
N	591
Mean (SD)	12.8 (6.0)
Current employment status
N	607
Not employed, *n* (%)	463 (76.3)
In paid employment, *n* (%)	109 (18.0)
Other (e.g., on sick leave), *n* (%)	35 (5.8)
Number of hours of work reduced (per week) over past 12 months in those who reported being in paid employment
N	106
0 (no reduction)	73 (68.9)
< 5 h, *n* (%)	10 (9.4)
5–10 h, *n* (%)	10 (9.4)
11–15 h, *n* (%)	5 (4.7)
16–20 h, *n* (%)	2 (1.9)
> 20 h, *n* (%)	6 (5.7)
Early retirement
N	444
Retired early, *n* (%)	164 (36.9)
Retired early due to PD, *n* (%)	126 (28.4)
Retired early but PD was not the main reason, *n* (%)	38 (8.6)
Did not retire early, *n* (%)	264 (59.5)
Prefer not to say, *n* (%)	16 (3.6)
Reduction in hours of daily activities (per week) over past 12 months
N	580
0 (no reduction)	220 (37.9)
< 5 h, *n* (%)	112 (19.3)
5–10 h, *n* (%)	86 (14.8)
11–15 h, *n* (%)	45 (7.8)
16–20 h, *n* (%)	23 (4.0)
> 20 h, *n* (%)	94 (16.2)

HRQoL, health-related quality of life; IQR, interquartile range; NMSQuest, Non-Motor Symptoms Questionnaire; PD, Parkinson’s disease; PDQ-39, Parkinson’s Disease Questionnaire-39; SD, standard deviation.

People with PD also had a wide range of non-motor symptoms and the mean (standard deviation [SD]) NMSQuest score was 12.8 (6.0). Non-motor symptoms reported by≥50% of participants comprised urgency of micturition (70.8%), nocturia (62.1%), feeling sad (61.8%), difficulty sleeping (59.7%), constipation (58.8%), forgetfulness (56.5%), difficulty concentrating (56.2%), loss of/change in taste or smell (54.7%), unpleasant leg sensations at rest (53.2%), high/low sexual interest (51.5%) and feeling anxious (50.0%).

The majority (76.3%) of participants were not working and 28.4% of the total population had retired early due to PD (Table 2). Among the 23.7% of participants who were working, 31.1% reported reducing work hours in the previous 12 months. The majority (62.1%) reported a reduced time spent on daily activities, such as shopping and gardening, during the previous 12 months, and a reduction of > 20 hours per week was reported by 16.2% of participants.

**Fig. 4:**
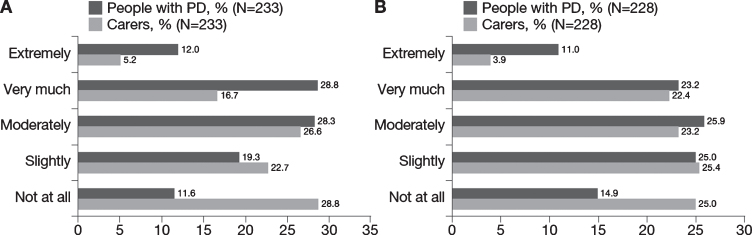
Impact of PD on relationships: A) Impact of PD on relationships for people with PD and matched carers and B) Change in impact on people with PD and matched carers as PD progressed. N reflects the total number of people with PD whose carers also answered questions regarding the impact PD has had on their relationship (“Has your relationship suffered because of the illness?” [left] and “Do you feel the impact of Parkinson’s on your relationship has changed as the disease has progressed?” [right]). N excludes missing values, “I don’t know” and “prefer not to say”. PD, Parkinson’s disease.

Approximately three-quarters (74.6%) of men reported problems sustaining a penile erection and 60.6% of women reported problems with orgasm. Participants also reported that PD affects domestic relationships: approximately 70% reported that PD had adversely affected family relationships moderately (28.3%), very much (28.8%) or extremely (12.0%), and approximately 60% reported that this had increased moderately (25.9%), very much (23.2%) or extremely (11.0%) as PD had progressed (Fig. 4).

### Impact of PD on impulse control behaviours

**Fig. 5:**
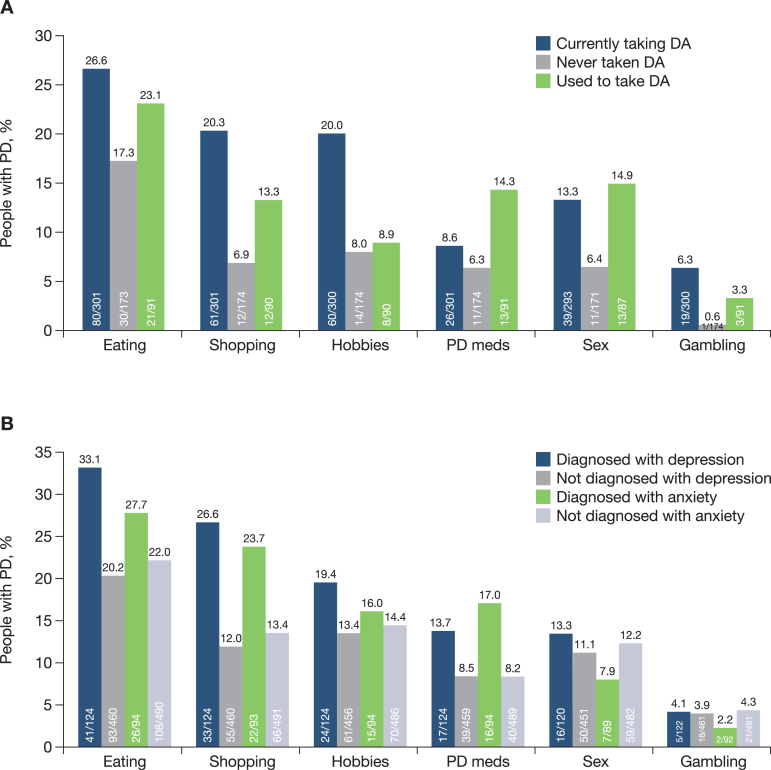
A) Impulse control behaviour by dopamine agonist usage and B) Impulse control behaviour in people with PD diagnosed with comorbid depression and anxiety. DA, dopamine agonist; PD, Parkinson’s disease.

Approximately 45% of people with PD had at least one impulse control behaviour, including binge eating (23.2%), compulsive shopping (15.1%), hobbyism (14.7%), hypersexuality (11.7%), compulsive consumption of PD medications (9.4%) and pathological gambling (4.2%). All impulse control behaviours were more frequently reported in those participants taking dopamine agonists compared with those who had never taken a dopamine agonist (Fig. 5A). Most impulse control behaviours were also more frequently reported in those taking dopamine agonists compared with those who had stopped taking dopamine agonists (Fig. 5A). People with PD diagnosed with depression (21.9% of the study population) or anxiety (15.8% of the study population) were more likely to report impulse control behaviours relating to eating, shopping, hobbyism and compulsive consumption of PD medications than those without these co-morbidities (Fig. 5B).

### Healthcare and social care resource utilisation

During the preceding 12 months, 96.0% of people with PD were under specialist care, 66.0% had accessed physiotherapy services and 24.0% had used mental health services. Overall, 26% of participants reported at least one emergency department presentation in the previous 12 months and 18% reported hospital admissions. Falls were the most common reason for emergency department presentation (30.0% of presentations) and hospital admission (13.2% of admissions). The majority of participants (approximately 90%) did not report routine use of community services (social care, paid caregiver, nursing care, overnight assistance, day care).

### Characteristics of carers of people with PD

Most of the carers were female (64.8%) and the partner/spouse of the person with PD (82.1%) (Table 3). The majority (76.8%) of the carers were aged between 45 and 74 years.

**Table 3:** Characteristics of carers of people with PD

Characteristic	N = 256
Country, *n* (%)
N	256
France	24 (9.4)
Germany	9 (3.5)
Italy	81 (31.6)
Portugal	30 (11.7)
Spain	38 (14.8)
United Kingdom	74 (28.9)
Gender
N	256
Male, *n* (%)	90 (35.2)
Female, *n* (%)	166 (64.8)
Age group
N	254
< 18–44 y, *n* (%)	19 (7.5)
45–54 y, *n* (%)	38 (15.0)
55–64 y, *n* (%)	65 (25.6)
65–74 y, *n* (%)	92 (36.2)
75–84 y, *n* (%)	38 (15.0)
≥85 y, *n* (%)	2 (0.8)
Relationship to person with PD
N	251
Partner/spouse, *n* (%)	206 (82.1)
Sibling, *n* (%)	35 (13.9)
Parent, *n* (%)	8 (3.2)
Child, *n* (%)	2 (0.8)

PD, Parkinson’s disease.

### Impact of caring for people with PD

Carers reported spending a mean 22.5 hours/week caring for the person with PD (Table 4) and the majority (55%) received no additional assistance from other family member or other sources. Overall, carers reported mild to moderate burden (mean [SD] ZBI total score, 26.6 [17.6]). Approximately 50% of carers reported that PD impacts their family relationships moderately (26.6%), very much (16.7%) or extremely (5.2%), and approximately 50% reported that this impact had increased moderately (23.2%), very much (22.4%) or extremely (3.9%) as the person with PD’s condition progressed (Fig. 4). Forty-six percent of carers reported that their partner’s PD had affected their sexual relationship.

**Table 4:** Burden of carers of people with PD

Parameter	N = 256
Hours of care to person with PD/week
N	214
Mean (SD)	22.5 (24.6)
Median (IQR)	14 (3–36)
ZBI total score^a^
N	246
Mean (SD)	26.6 (17.6)
Median (IQR)	25 (11–39)
Burden severity by ZBI total score^a^
N	246
Severe (ZBI total score > 60), *n* (%)	7 (2.8)
Moderate/severe (ZBI total score 41–60), *n* (%)	48 (19.5)
Mild/moderate (ZBI score 21–40), *n* (%)	84 (34.1)
Little/no burden (ZBI≤20), *n* (%)	107 (43.5)
Assistance from others in caring for person with PD
N	242
Family member, *n* (%)	72 (29.8)
Friend, *n* (%)	32 (13.2)
Paid nurse, *n* (%)	8 (3.3)^b^
Other paid caregiver, *n* (%)	29 (12.0)^b^

^a^Assessed over previous month. ^b^N = 241. IQR, interquartile range; PD, Parkinson’s disease; SD, standard deviation; ZBI, Zarit Burden Inventory.

## DISCUSSION

The PRISM study provides information on the disease burden and treatment of people with PD. A range of co-morbidities were reported, consistent with previous reports [21, 22]. The rate of hypertension observed in PRISM (25.3%) was lower than what might be expected, since the overall prevalence of hypertension in adults has been estimated at 30–45% increasing to > 60% in people aged > 60 years [23], and previous studies in people with PD have also reported a higher figure than that observed in PRISM (e.g., 41.1% in a study of a large Scottish primary care database [22]). The relatively low rate in PRISM might have been due to under-reporting among participants with well-controlled blood pressure. Previous evidence of a potential association between Type 2 diabetes and PD [24] was not supported by the current study.

Levodopa was currently used (last 12 months) by the majority of respondents (∼90%), with 22% taking it as monotherapy. Only a small proportion of participants were currently using dopamine agonists and MAO-B inhibitors as monotherapy (4% and 2%, respectively), considerably lower than reported in earlier studies [25–27]. In one survey of 500 people with PD from the USA and five European countries (France, Germany, Italy, Spain and the United Kingdom), which was conducted during 2003–2004, 71% of early-stage patients were being treated with monotherapy, of whom 39% were taking dopamine agonists [25]. In the Spanish, multicentre, retrospective ROPI-PARK study (published in 2009), which evaluated the use of ropinirole in approximately 420 people with PD, 24% had been treated with dopamine agonist monotherapy in the previous 18 months [26]. In another study, conducted in the United Kingdom between 2004 and 2015, 21% of over 6000 people with PD treated with anti-PD medication were taking ropinirole monotherapy and a further 17% were taking pergolide monotherapy, over a median follow-up duration of 2.8 years [27]. The lower use of dopamine agonist monotherapy in PRISM may reflect changes in treatment recommendations and prescribing practice over time, since dopamine agonists and MAO-B inhibitors were preferred over levodopa as initial monotherapy options 25 years ago because of their perceived potential to delay the onset of dyskinesia and/or motor fluctuations, and a misplaced notion that they were neuroprotective [28].

There was considerable variation between countries in terms of therapeutic regimens, which may reflect cultural differences in prescribing practice, but may also reflect the differences in the disease stage of patient populations between individual countries. For example, although the use of levodopa monotherapy was highest in the United Kingdom, a higher proportion of participants had been diagnosed within the past 5 years, compared with the other countries (55% in the United Kingdom, 47% in Spain, 42% in Portugal, 41% in Italy and 33% in both Germany and France). Given the range of therapies available for PD and the long duration of disease, therapeutic regimens are tailored for the individual patient based in part on the most disabling symptoms of the disease (including both motor and non-motor symptoms and motor fluctuations); individual preferences of people with PD may also influence treatment decisions. Although PD severity (disease stage) was not measured in PRISM, further analyses of medication use in relation to disease duration, age and symptomatology will allow for clearer conclusions about treatment patterns and differences between countries. For instance, there was a trend for a lower percentage of levodopa users in the younger versus older age categories.

Impulse control behaviours were reported by approximately 45% of people with PD and these were more frequently reported in those currently taking dopamine agonists than in those who had never taken, or stopped taking, a dopamine agonist. The prevalence of impulse control behaviours in the PRISM population appears to be higher than in other similar studies, where a prevalence of up to approximately 35% has been reported [29]. However, a 5-year longitudinal study conducted in France, in which impulse control behaviours were evaluated by movement disorders specialists during face-to-face semi-structured interviews, reported a cumulative incidence of 46% in a population of over 300 patients with PD who did not have impulse control behaviours at baseline, and a cumulative incidence of 52% in those who had ever used dopamine agonists [30]. The prospective, non-interventional, multicentre ICARUS study (Impulse Control disorders And the association of neuRopsychiatric symptoms, cognition and qUality of life in ParkinSon disease) assessed the presence of impulse control disorders/other compulsive behaviours (‘ICD behaviours’) in over 1000 people with PD over a 2-year period. Point prevalence of ICD behaviours remained stable during follow-up, being 29% at baseline, 29% at year 1 and 27% at year 2 [3]. In ICARUS, the most prevalent type of ICD behaviour was compulsive eating, followed by punding (a need to carry out a pointless repetitive motor behaviour over long periods of time), compulsive sexual behaviour, gambling and shopping [3]. In PRISM, eating was also the most commonly reported impulse control behaviour, followed by shopping and hobbyism. In ICARUS, people with PD with ICD behaviour were shown to have more severe depression, poorer sleep quality and reduced quality of life, compared with those who did not have ICD behaviours [3]. In PRISM, there was also an apparent association between diagnosis of depression and/or anxiety and higher rates of most reported impulse control behaviours. Several patient factors have been found to be associated with the development of impulse control behaviours in those treated with dopamine agonists, including a history of psychiatric symptoms, earlier onset of disease, longer disease duration, dopamine agonist dosage, male sex, younger age, and motor complications in PD [29].

The median PDQ-39 summary score was 29.1; however, the IQR was 18.0–43.9, indicating that there was variability between individuals in the degree to which PD impacts their HRQoL. The PDQ-39 results indicate that HRQoL was particularly affected by problems with bodily discomfort (median score, 41.7) and mobility (median score, 35.0). People with PD diagnosed before age 50 years were shown to have worse HRQoL scores than those diagnosed after age 50 years, as were those diagnosed with anxiety and/or depression in comparison with those not diagnosed with either condition. These findings are consistent with those of a study conducted in 817 people with PD from France, Germany, Italy, Spain, and the United Kingdom (mean age, 66.5 years; 54% male; mean disease duration, 3.3 years), in which the mean PDQ-39 summary score was 25.4 [31]. As in PRISM, the mobility domain was particularly impaired (mean score, 36.7) but the bodily discomfort score was lower than in PRISM (mean score, 24.7) [31]. In another European study, in which the PDQ-39 was completed by a postal survey (*n* = 202; mean age, 69.8 years; mean disease duration, 8.7 years), mobility (median score, 45) and bodily discomfort (median score, 41.7) were also the domains that were most affected [32]. The Italian multicentre, naturalistic PaRkInson And non-MOtor symptoms (PRIAMO) study investigated the prevalence of non-motor symptoms in 1072 people with PD (mean age, 67.4 years; 60% male; mean disease duration, 5.1 years) [33] and used the PDQ-39 to prospectively assess the impact of non-motor symptoms on HRQoL in a subset of 377 people with PD over 2 years [34]. Although there was no overall change in the mean PDQ-39 summary score over this time period, the summary score significantly increased (indicating worsening HRQoL) in patients who developed non-motor symptoms in the cardiovascular, apathy, psychiatric and fatigue domains during the 24-month study period, compared with patients who experienced regression of the same symptoms in these domains (*p* < 0.0045 for all comparisons) [34]. Taken together, these findings indicate that although non-motor symptoms contribute significantly to reduced quality of life, motor disability due to bradykinesia and rigidity is, for most patients, the most important factor contributing to reduced quality of life.

People with PD in PRISM had a high incidence and wide range of non-motor symptoms, including urinary difficulties, constipation, loss of/change in taste or smell, sleeping difficulties, feelings of sadness, anxiety, problems with forgetfulness and difficulties concentrating. The mean±SD NMSQuest score (12.8±6.0) is compatible with several earlier studies: 9.3±4.3 (Italy); 11.0±5.3 (Germany); and 10.0±5.3 (United Kingdom) [35]. Non-motor symptoms may be present in the early stages of PD and increase in frequency and severity as the disease progresses, impairing HRQoL and overall health status [36, 37]. Non-motor symptoms are also strongly associated with the need for residential care, with one report claiming that 80% of people with PD have dementia 20 years after diagnosis [37].

Over three-quarters of participants in the PRISM study were not working and > 60% reported a reduced time spent on daily activities during the previous 12 months. Although the age profile of the population (mean age, 65 years) indicated that many may have been coming towards the end of their working lives, 28% had retired early due to PD and almost a third of those who had not retired reported that they had reduced their work hours in the previous 12 months. In a Swedish population-based cohort study in which > 1400 people with PD (median age, 63 years) completed a postal questionnaire, only 24% of people with PD were employed≥10 years after diagnosis and only 6% worked full-time [38]. Moreover, compared with matched controls, unemployment status independently correlated with a greater risk of dissatisfaction with life (*p* < 0.05) [38]. In another questionnaire-based study of 937 working-aged people with PD who were members of the Finnish Parkinson Association (median age, 59 years), only 150 (16%) were still working (full-time, 12%; part-time, 4%) [39]. In line with the PRISM population, 37% of people with PD in the Finnish study had retired early due to PD; the median age at retirement was 53.4 years and the median working time after an established PD diagnosis was 1.7 years (4.3 years for those in part-time work) [39]. In the PRISM study, approximately 70% of people with PD reported that PD impacted their family relationships; a disturbance that worsened with increased duration of disease. The high rate of sexual problems reported by people with PD warrants further study.

PRISM also provided insights into the impact of PD on the use of health and social care resources. In the past 12 months, almost all people with PD (96%) were under specialist care, more than one quarter reported at least one hospital emergency department presentation, and approximately one fifth reported an inpatient admission. These findings illustrate the burden of PD on society and will be the focus of further research using data from PRISM.

Although carers reported mild to moderate burden (mean ZBI score, 26.6), almost all (~90%) reported that PD had impacted on family relationships and almost 50% reported that caring for a person with PD had adversely affected their sexual relationship. These findings are consistent with those of other studies. For example, in an Italian study of 126 patients (mean age, 69 years) and their carers (mean age, 58 years), the majority of carers were women (70%) and spouse to the person with PD (60%), although 32% of patients were cared for by sons/daughters [12]. Most carers (92%) had been caring for the person with PD for≥12 months, and over half (53%) were caregiving 24 h a day. Carers of people with PD who were receiving standard of care (as opposed to a continuous dopaminergic delivery system) had a mean ZBI score of 31.4, indicating mild to moderate burden (as in PRISM) [12].

Since the survey was made available primarily via patient groups’ online networks and at the discretion of selected healthcare centres, this might have resulted in a study population that was not necessarily representative of the general PD population; for example, ethnicity was not recorded as part of the survey, so it was not possible to determine whether minority ethnic groups were appropriately represented. Moreover, online survey methods are likely to select a younger and more educated population. The poor and the very old are two groups that are likely to have been underrepresented (only 8% of the PRISM population were aged≥80 years). People with advanced PD were also underrepresented, since the median disease duration of people with PD was 6 years. The study depended on questionnaires and did not permit formal neurological assessment, assessment of the severity of motor disability, or objective evaluation of impulse control behaviours using a structured interview; in addition, the reporting of co-morbidities was based on patient-reported diagnoses, rather than the retrieval of objective information from medical records. Online survey methods do, however, have the advantages of allowing data collection in hard-to-reach populations and in those unable to travel to medical centres (for example, due to restrictions imposed by COVID-19). In people with PD who provided a partial response to the survey, the questions that were not answered tended to be towards the end of survey (for example, 261/262 replied to the PDQ-39 compared with 8/262 to Q81 and 4/262 to Q83), emphasising the importance of positioning the key questions in any study at the beginning.

Although the findings presented here are descriptive in nature, the size of the population is a strength of the study, which will allow further statistical examination of the data in the future (for example, multivariate analyses to explore drivers of HRQoL impairment and carer burden; analyses to investigate the impact of disease duration on characteristics such as use of PD medication, sexual functioning and impulse control behaviours). It is anticipated that future country-specific analyses will be conducted using the data collected in PRISM. PRISM also offers the opportunity to analyse between-country differences for issues such as medication prescribing practices and the role of allied health services.

The PRISM study sheds further light on the lives of people with PD, highlighting the many challenges they face, including the high rates of comorbidity, motor and non-motor symptoms and impulse control disorders that may adversely affect ability to work/perform daily activities and quality of life. The findings also provide information on how medical treatment approaches vary considerably between countries across Europe. Finally, PRISM demonstrates that the wellbeing of those who care for people with PD is also adversely affected and needs to receive greater recognition from society.

## DATA AVAILABILITY

BIAL is committed to help improving the care of PD patients through high-quality scientific research. The full results dataset will be made available for further analyses to any health care professional or academic researcher at https://prism.bial.com/.

## Supplementary Material

Supplementary Material
