# Supplementary material for: The Parkinson’s Real-World Impact Assessment (PRISM) Study: A European Survey of the Burden of Parkinson’s Disease in Patients and their Carers
Source: J Parkinsons Dis. 2021 Aug 2;11(3):1309–23. doi: 10.3233/JPD-212611 (PMC8461669; doi:10.3233/JPD-212611)
Supplement: Supplementary Material [file jpd-11-jpd212611-s001.pdf]

Supplementary Material

The Parkinson’s Real-World Impact Assessment (PRISM) Study: A European Survey of the Burden of Parkinson’s Disease in Patients and their Carers

Supplementary Material 1. PRISM survey

PART 1: PWP survey
Some details about the person with Parkinson's
Your responses to these questions will help us to interpret your responses to the other questions. All of the information that you give will be kept confidential.
1. Which country do you live in? *

- ☐ England
- ☐ Scotland
- ☐ Wales
- ☐ Northern Ireland
- ☐ France
- ☐ Germany
- ☐ Italy
- ☐ Portugal
- ☐ Spain
- ☐ Other (please specify below)

If your country is not listed, please specify below.

Your responses to these questions will help us to interpret your responses to the other questions. All of the information that you give will be kept confidential.

2. How old are you (in years)? *

3. How old were you when you were first diagnosed with Parkinson's? If you can't remember, please make your best estimate. *

Please enter the age you were diagnosed if you were under 50 or over 90

4. What is your gender? *

- ☐ Male

- [ ] Female
- [ ] Other
- [ ] Prefer not to say

5. What is the highest level of formal education that you have completed? *

- [ ] Primary or secondary school/vocational level 1 & 2/trade apprenticeship
- [ ] Secondary school advanced or vocational level 3
- [ ] Further education or training college below degree level
- [ ] Some university
- [ ] Completed university degree
- [ ] Post-graduate degree
- [ ] Prefer not to say

6. How far do you travel (one-way) from your home to a specialist centre or a specialist consultant for Parkinson's disease treatment (neurologist)? *

- [ ] Less than 30 miles / 50km
- [ ] 30-60 miles / 50-100km
- [ ] More than 60 miles (100km)
- [ ] Unknown

7. In addition to Parkinson's disease, are you currently being treated for, or have you been diagnosed (current diagnosis) with, any of the following...? Check all that apply *

- [ ] Heart issues (heart attack, heart failure)
- [ ] Stroke
- [ ] Peripheral vascular disease (also known as peripheral arterial disease)
- [ ] High blood pressure
- [ ] Asthma
- [ ] Chronic obstructive pulmonary disease (COPD)
- [ ] Diabetes (type 1 or type 2)

- [ ] Kidney disease
- [ ] Liver disease
- [ ] Gastritis (ulcer)
- [ ] Cancer
- [ ] Dementia
- [ ] Rheumatic diseases (inflammatory arthritis, autoimmune diseases such as lupus and vasculitis, bone conditions such as osteoarthritis and osteoporosis)
- [ ] Depression
- [ ] Anxiety
- [ ] I have no other diagnosis
- [ ] Prefer not to say
- [ ] Other (please specify):

Parkinson's Disease Quality of Life Questionnaire (PDQ-39)

PDQ-39 © Copyright, Oxford University Innovation Limited 1993. All Rights Reserved. The authors, being Professor Crispin Jenkinson, Professor Ray Fitzpatrick and Ms Viv Peto, have asserted their moral rights.

Please select one response option for every question Due to having Parkinson's disease, How often during the last month have you... Had difficulty doing the leisure activities which you would like to do? *

- [ ] Never
- [ ] Occasionally
- [ ] Sometimes
- [ ] Often
- [ ] Always or cannot do at all

Due to having Parkinson's disease, How often during the last month have you... Had difficulty looking after your home, e.g. DIY, housework, cooking? *

- [ ] Never
- [ ] Occasionally
- [ ] Sometimes
- [ ] Often

- [ ] Always or cannot do at all

Due to having Parkinson’s disease, How often during the last month have you... Had difficulty carrying bags of shopping? *

- [ ] Never
- [ ] Occasionally
- [ ] Sometimes
- [ ] Often
- [ ] Always or cannot do at all

Due to having Parkinson’s disease, How often during the last month have you... Had problems walking half a mile? *

- [ ] Never
- [ ] Occasionally
- [ ] Sometimes
- [ ] Often
- [ ] Always or cannot do at all

Due to having Parkinson’s disease, How often during the last month have you... Had problems walking 100 yards? *

- [ ] Never
- [ ] Occasionally
- [ ] Sometimes
- [ ] Often
- [ ] Always or cannot do at all

Due to having Parkinson’s disease, How often during the last month have you... Had problems getting around the house as easily as you would like? *

- [ ] Never
- [ ] Occasionally
- [ ] Sometimes
- [ ] Often

- [ ] Always or cannot do at all

Due to having Parkinson's disease, How often during the last month have you... Had difficulty getting around in public? *

- [ ] Never
- [ ] Occasionally
- [ ] Sometimes
- [ ] Often
- [ ] Always or cannot do at all

Due to having Parkinson's disease, How often during the last month have you... Needed someone else to accompany you when you went out? *

- [ ] Never
- [ ] Occasionally
- [ ] Sometimes
- [ ] Often
- [ ] Always or cannot do at all

Due to having Parkinson's disease, How often during the last month have you... Felt frightened or worried about falling over in public *

- [ ] Never
- [ ] Occasionally
- [ ] Sometimes
- [ ] Often
- [ ] Always

Due to having Parkinson's disease, How often during the last month have you... Been confined to the house more than you would like? *

- [ ] Never
- [ ] Occasionally
- [ ] Sometimes

- [ ] Often
- [ ] Always

Due to having Parkinson's disease, How often during the last month have you... Had difficulty washing yourself? *

- [ ] Never
- [ ] Occasionally
- [ ] Sometimes
- [ ] Often
- [ ] Always

Due to having Parkinson's disease, How often during the last month have you... Had difficulty dressing yourself? *

- [ ] Never
- [ ] Occasionally
- [ ] Sometimes
- [ ] Often
- [ ] Always

Due to having Parkinson's disease, How often during the last month have you... Had problems doing up your shoe laces? *

- [ ] Never
- [ ] Occasionally
- [ ] Sometimes
- [ ] Often
- [ ] Always

Due to having Parkinson's disease, How often during the last month have you... Had problems writing clearly? *

- [ ] Never
- [ ] Occasionally
- [ ] Sometimes

- [ ] Often
- [ ] Always

Due to having Parkinson's disease, How often during the last month have you... Had difficulty cutting up your food? *

- [ ] Never
- [ ] Occasionally
- [ ] Sometimes
- [ ] Often
- [ ] Always

Due to having Parkinson's disease, How often during the last month have you... Had difficulty holding a drink without spilling it? *

- [ ] Never
- [ ] Occasionally
- [ ] Sometimes
- [ ] Often
- [ ] Always

Due to having Parkinson's disease, How often during the last month have you... Felt depressed? *

- [ ] Never
- [ ] Occasionally
- [ ] Sometimes
- [ ] Often
- [ ] Always

Due to having Parkinson's disease, How often during the last month have you... Felt isolated and lonely? *

- [ ] Never
- [ ] Occasionally
- [ ] Sometimes

- [ ] Often
- [ ] Always

Due to having Parkinson’s disease, How often during the last month have you... Felt weepy or tearful? *

- [ ] Never
- [ ] Occasionally
- [ ] Sometimes
- [ ] Often
- [ ] Always

Due to having Parkinson’s disease, How often during the last month have you... Felt angry or bitter? *

- [ ] Never
- [ ] Occasionally
- [ ] Sometimes
- [ ] Often
- [ ] Always

Due to having Parkinson’s disease, How often during the last month have you... Felt anxious? *

- [ ] Never
- [ ] Occasionally
- [ ] Sometimes
- [ ] Often
- [ ] Always

Due to having Parkinson’s disease, How often during the last month have you... Felt worried about your future? *

- [ ] Never
- [ ] Occasionally
- [ ] Sometimes

- [ ] Often
- [ ] Always

Due to having Parkinson’s disease, How often during the last month have you... Felt you had to conceal your Parkinson's from people? *

- [ ] Never
- [ ] Occasionally
- [ ] Sometimes
- [ ] Often
- [ ] Always

Due to having Parkinson’s disease, How often during the last month have you... Avoided situations which involve eating or drinking in public? *

- [ ] Never
- [ ] Occasionally
- [ ] Sometimes
- [ ] Often
- [ ] Always

Due to having Parkinson’s disease, How often during the last month have you... Felt embarrassed in public due to having Parkinson's disease? *

- [ ] Never
- [ ] Occasionally
- [ ] Sometimes
- [ ] Often
- [ ] Always

Due to having Parkinson’s disease, How often during the last month have you... Felt worried by other people's reaction to you? *

- [ ] Never
- [ ] Occasionally

- [ ] Sometimes
- [ ] Often
- [ ] Always

Due to having Parkinson’s disease, How often during the last month have you... Had problems with your close personal relationships? *

- [ ] Never
- [ ] Occasionally
- [ ] Sometimes
- [ ] Often
- [ ] Always

Due to having Parkinson’s disease, How often during the last month have you... Lacked support in the ways you need from your spouse or partner? *

- [ ] Never
- [ ] Occasionally
- [ ] Sometimes
- [ ] Often
- [ ] Always
- [ ] If you do not have a spouse or partner, please tick here

Due to having Parkinson’s disease, How often during the last month have you... Lacked support in the ways you need from your family or close friends? *

- [ ] Never
- [ ] Occasionally
- [ ] Sometimes
- [ ] Often
- [ ] Always

Due to having Parkinson’s disease, How often during the last month have you... Unexpectedly fallen asleep during the day? *

- [ ] Never
- [ ] Occasionally
- [ ] Sometimes
- [ ] Often
- [ ] Always

Due to having Parkinson’s disease, How often during the last month have you... Had problems with your concentration, e.g. when reading or watching TV? *

- [ ] Never
- [ ] Occasionally
- [ ] Sometimes
- [ ] Often
- [ ] Always

Due to having Parkinson’s disease, How often during the last month have you... Felt your memory was bad? *

- [ ] Never
- [ ] Occasionally
- [ ] Sometimes
- [ ] Often
- [ ] Always

Due to having Parkinson’s disease, How often during the last month have you... Had distressing dreams or hallucinations? *

- [ ] Never
- [ ] Occasionally
- [ ] Sometimes
- [ ] Often
- [ ] Always

Due to having Parkinson’s disease, How often during the last month have you... Had difficulty with your speech? *

- [ ] Never
- [ ] Occasionally
- [ ] Sometimes
- [ ] Often
- [ ] Always

Due to having Parkinson's disease, How often during the last month have you... Felt unable to communicate with people properly? *

- [ ] Never
- [ ] Occasionally
- [ ] Sometimes
- [ ] Often
- [ ] Always

Due to having Parkinson's disease, How often during the last month have you... Felt ignored by people? *

- [ ] Never
- [ ] Occasionally
- [ ] Sometimes
- [ ] Often
- [ ] Always

Due to having Parkinson's disease, How often during the last month have you... Had painful muscle cramps or spasms *

- [ ] Never
- [ ] Occasionally
- [ ] Sometimes
- [ ] Often
- [ ] Always

Due to having Parkinson's disease, How often during the last month have you... Had aches and pains in your joints or body? *

- [ ] Never
- [ ] Occasionally
- [ ] Sometimes
- [ ] Often
- [ ] Always

Due to having Parkinson’s disease, How often during the last month have you... Felt unpleasantly hot or cold? *

- [ ] Never
- [ ] Occasionally
- [ ] Sometimes
- [ ] Often
- [ ] Always

Pharmaceutical treatment for Parkinson's
This part of the survey is for the person with Parkinson's. Your answers could help improve future treatments.

8. Did your first prescribed oral medication for Parkinson’s contain levodopa? This includes: Co-careldopa (brand names: Caramet CR, Sinemet, Sinemet plus, Lecado) Benserazide (brand name: Madopar) Entacapone and co-careldopa (brand name: Stalevo) Mucuna pruriens *

- [ ] Yes
- [ ] No
- [ ] Don't know
- [ ] Prefer not to say

9. How long after you were diagnosed with Parkinson's were you first prescribed a therapy containing levodopa? *

- [ ] Within the first year
- [ ] 1-2 years after diagnosis
- [ ] 3-4 years after diagnosis
- [ ] 5 or more years after diagnosis
- [ ] I have never used levodopa

☐ Prefer not to say

10. Which oral medications(s) are you currently taking (in the past 12 months) or did you take in the past (more than 12 months ago) for Parkinson's? If you stopped using these medications, what was your reason? Please select an answer from the drop down menu for each group of medications. *

Levo-dopa<br>Co-careldopa (brand names: Caramet CR, Sinemet, Sinemet plus, Lecado)<br>Benserazide (brand name: Madopar)<br>Entacapone and co-careldopa (brand name: Stalevo)	I currently take (in the past 12 months)<br>I have never taken these drugs<br>Used to take, but they have stopped working for me<br>Used to take, but they have had an adverse reaction<br>Used to take, but had a problem wearing off<br>Used to take, I don't know why my doctor changed my prescription<br>Other (please describe in comments below)<br>I prefer not to say
Dopamine agonists<br>Pramipexole (brand names: Mirapex, Mirapexin, Sifrol)<br>Ropinirole (brand name: Requip XL)<br>Rotigotine (brand name: Neupro)<br>Bromocriptine (brand name: Parlodel)<br>Cabergoline (brand names: Cabaser, Dostinex)<br>Pergolide (brand names: Permax, Prascend)<br>Piribedil (brand names: Trivastal, Pronoran, Clarium)<br>Apomorphine (brand name: APO-go)	I currently take (in the past 12 months)<br>I have never taken these drugs<br>Used to take, but they have stopped working for me<br>Used to take, but they have had an adverse reaction<br>Used to take, but had a problem wearing off<br>Used to take, I don't know why my doctor changed my prescription<br>Other (please describe in comments below)<br>I prefer not to say
COMT inhibitors<br>Entacapone (brand name: Comtan)<br>Tolcapone (brand name: Tasmar)<br>Opicapone (brand name: Ongentys)	I currently take (in the past 12 months)<br>I have never taken these drugs<br>Used to take, but they have stopped working for me<br>Used to take, but they have had an adverse reaction<br>Used to take, but had a problem wearing off<br>Used to take, I don't know why my doctor changed my prescription<br>Other (please describe in comments below)<br>I prefer not to say
MAO-B inhibitors<br>Rasagiline (brand name: Azilect)<br>Selegiline hydrochloride (brand names: Eldepryl, Selgene, Apo-selegilin)<br>Safinamide (brand name: Xadago)	I currently take (in the past 12 months)<br>I have never taken these drugs<br>Used to take, but they have stopped working for me<br>Used to take, but they have had an adverse reaction<br>Used to take, but had a problem wearing off<br>Used to take, I don't know why my doctor changed my prescription<br>Other (please describe in comments below)<br>I prefer not to say
Anticholinergics<br>Orphenadrine (brand name: Norflex)<br>Procyclidine (brand name: Kemadrin)<br>Trihexyphenidyl (brand names: Benzhexol, Artane, and Trihex)	I currently take (in the past 12 months)<br>I have never taken these drugs<br>Used to take, but they have stopped working for me<br>Used to take, but they have had an adverse reaction<br>Used to take, but had a problem wearing off<br>Used to take, I don't know why my doctor changed my prescription<br>Other (please describe in comments below)<br>I prefer not to say

Amantadine (brand name: Symmetrel)	I currently take (in the past 12 months)<br>I have never taken these drugs<br>Used to take, but they have stopped working for me<br>Used to take, but they have had an adverse reaction<br>Used to take, but had a problem wearing off<br>Used to take, I don’t know why my doctor changed my prescription<br>Other (please describe in comments below)<br>I prefer not to say
Rivastigmine (brand name: Exelon)	I currently take (in the past 12 months)<br>I have never taken these drugs<br>Used to take, but they have stopped working for me<br>Used to take, but they have had an adverse reaction<br>Used to take, but had a problem wearing off<br>Used to take, I don’t know why my doctor changed my prescription<br>Other (please describe in comments below)<br>I prefer not to say

If you selected other, please describe the reason below.

11. Do you take generic medicines when these are available? *

- [ ] Always
- [ ] Most of the time
- [ ] Sometimes
- [ ] Never
- [ ] Don't know
- [ ] Prefer not to say

12. Have you ever taken part in a clinical trial for a new treatment for Parkinson's? *

- [ ] Yes, I am currently
- [ ] Yes, in the past
- [ ] No, but I would like to
- [ ] No, and I would not like to
- [ ] Prefer not to say

13. What is the name of the experimental drug or treatment? *

Out-of-pocket costs for Parkinson's medication
This part of the survey is for the person with Parkinson's.

14. Do you pay out of your own pocket for medicines, vitamins or supplements to treat Parkinson’s symptoms? *

- Yes
- No
- I don't know
- Prefer not to say

15. In the past 3 months, approximately how much have you spent out of your own pocket on medicines, vitamins and supplements used to treat Parkinson's symptoms? Please do not include medicines, vitamins or supplements that you take for other reasons *

	£0	<£10/month	£10-£50/month	£51-£100/month	£101-200/month	>£200/month	Don't know	Prefer not to say
Prescribed medicines								
Medicines without prescription (over the counter)								
Vitamins/supplements								

16. Please enter the names of any medications, vitamins and supplements that you have paid for (with your own money) in the last 3 months to treat Parkinson's symptoms.

	Medication, vitamin or supplement
1	
2	
3	
4	
5	
6	
7	
8	

Medication, vitamin or supplement

9

10

If more than 10, please include details here

Specialist

This part of the survey is for the person with Parkinson's. Your answers may help to improve services.

17. In the past 12 months, have you seen a specialist consultant (e.g. neurologist, geriatrician)? *

- Yes
- No
- Prefer not to say

18. In the past 12 months, how many times have you seen a specialist consultant (e.g. neurologist, geriatrician)? *

Please select the number of times (in the past 12 months)

19. In the past 12 months, did you pay for a specialist consultant (e.g. neurologist, geriatrician)? *

- I paid the full cost
- I paid some
- I did not pay
- I don't know
- Prefer not to say

20. In the past 12 months, how much did you pay for one visit to a specialist consultant (e.g. neurologist, geriatrician)? (£/GBP) *

GP/Family Doctor

This part of the survey is for the person with Parkinson's. Your answers may help to improve services.

21. In the past 12 months, have you seen a General Practitioner (GP) or Family Doctor? *

- [ ] Yes
- [ ] No
- [ ] Prefer not to say

22. In the past 12 months, how many times have you seen a General Practitioner (GP) or Family Doctor? *

Please select the number of times (in the past 12 months) [ ]

23. In the past 12 months, did you pay for a General Practitioner (GP) or Family Doctor? *

- [ ] I paid the full cost
- [ ] I paid some
- [ ] I did not pay
- [ ] I don't know
- [ ] Prefer not to say

24. In the past 12 months, how much did you pay for one visit to a General Practitioner (GP) or Family Doctor? (£/GBP) *

[ ]

Physiotherapy
This part of the survey is for the person with Parkinson's. Your answers may help to improve services.

25. In the past 12 months, have you seen a physiotherapist? *

- [ ] Yes
- [ ] No
- [ ] Prefer not to say

26. In the past 12 months how many times have you seen a physiotherapist? *

Please select the number of times (in the past 12 months) [ ]

27. In the past 12 months, did you pay for a physiotherapist? *

- [ ] I paid the full cost
- [ ] I paid some
- [ ] I did not pay

- [ ] I don't know
- [ ] Prefer not to say

28. In the past 12 months, how much did you pay for one visit to a physiotherapist? (£/GBP) *

[ ]

Mental health services
This part of the survey is for the person with Parkinson's. Your answers may help to improve services.

29. In the past 12 months, have you used mental health services? (e.g. psychiatrist, psychologist or counsellor) *

- [ ] Yes, psychiatrist
- [ ] Yes, psychologist
- [ ] Yes, counsellor or therapist
- [ ] No
- [ ] Prefer not to say

30. In the past 12 months, how many times have you used mental health services? (e.g. psychiatrist, psychologist or counsellor etc.) *

Please select the number of times (in the past 12 months) [ ]

31. In the past 12 months, did you pay for mental health services? (e.g. psychiatrist, psychologist or counsellor) *

- [ ] I paid the full cost
- [ ] I paid some
- [ ] I did not pay
- [ ] I don't know
- [ ] Prefer not to say

32. In the past 12 months, how much did you pay for one mental health service visit? (e.g. psychiatrist, psychologist or counsellor) (£/GBP) *

[ ]

Other health services
This part of the survey is for the person with Parkinson's. Your answers may help to improve services.

33. In the past 12 months, did you use the services listed below? *

	How many times?	How much did you pay each visit? (£/GBP)
Primary care/general practice nurse		
Specialist Parkinson's disease nurse		
Occupational therapist		
Speech/language therapist		
Other (please specify below)		

Which other health service did you use? Please don't enter hospital visits here.

Accident & Emergency (A&E) Department
This part of the survey is for the person with Parkinson's. Your answers may help to improve services.

34. Did you attend an Accident & Emergency (A&E) Department in the last 12 months? *

- [ ] Yes
- [ ] No
- [ ] Prefer not to say

35. What was the reason for your first attendance? (If you attended more than once, you can enter information about other attendances next.) *

- [ ] Heart issues (heart attack, heart failure)
- [ ] Stroke
- [ ] High blood pressure
- [ ] Fall
- [ ] Chest infection (pneumonia)
- [ ] Skin infection
- [ ] I don't know
- [ ] Prefer not to say
- [ ] Other (please specify):

36. Did you pay out of your own pocket for the Accident & Emergency (A&E) Department attendance? *

- [ ] I paid full
- [ ] I paid some
- [ ] I did not pay
- [ ] I don't know
- [ ] Prefer not to say

37. How much did you pay for the Accident & Emergency (A&E) department attendance? (£/GBP) *

38. Did you have another Accident & Emergency (A&E) Department attendance in the last 12 months? *

- [ ] Yes
- [ ] No

39. What was the reason for your second attendance? *

- [ ] Heart issues (heart attack, heart failure)
- [ ] Stroke
- [ ] High blood pressure
- [ ] Fall
- [ ] Chest infection (pneumonia)
- [ ] Skin infection
- [ ] I don't know
- [ ] Prefer not to say
- [ ] Other (please specify):

40. Did you pay out of your own pocket for the Accident & Emergency (A&E) Department attendance? *

- [ ] I paid full
- [ ] I paid some
- [ ] I did not pay
- [ ] I don't know

☐ Prefer not to say

29. Accident & Emergency (A&E) Department
Please answer all of the questions on this page about the second attendance.

41. How much did you pay for the Accident & Emergency (A&E) Department attendance? (£/GBP) *

42. Did you have another Accident & Emergency (A&E) Department attendance in the last 12 months? *

☐ Yes

☐ No

43. Please list the reasons for any more emergency department attendances below - one admission per line.

	Reason	How much did you pay out of your own pocket? (£/GBP)
1		
2		
3		
4		
5		
6		
7		
8		
9		
10		

32. Hospital admissions

44. Have you been admitted to hospital in the last 12 months? *

☐ Yes

☐ No

☐ Prefer not to say

Hospital admissions
Please answer all of the questions on this page about one hospital stay. You can enter information about other hospital stays next.

45. What was the reason you were admitted? (If you went to hospital more than once, you can enter information about other attendances next.) *

- [ ] Heart issues (heart attack, heart failure)
- [ ] Stroke
- [ ] High blood pressure
- [ ] Fall
- [ ] Chest infection (pneumonia)
- [ ] Skin infection
- [ ] I don't know
- [ ] Prefer not to say
- [ ] Other (please specify): ____________________

How many nights did you stay in hospital? *

If more than 10 nights, enter the number below.

____

46. Did you pay out of your own pocket for the hospital admission? *

- [ ] Yes, I paid full
- [ ] Yes, I paid some
- [ ] No
- [ ] I don't know
- [ ] Prefer not to say

47. How much did you pay? (£/GBP) *

____

48. Did you have another hospital admission in the last 12 months? *

- [ ] Yes
- [ ] No

49. What was the reason you were admitted? *

- [ ] Heart issues (heart attack, heart failure)
- [ ] Stroke
- [ ] High blood pressure
- [ ] Fall
- [ ] Chest infection (or pneumonia)
- [ ] Skin infection
- [ ] I don't know
- [ ] Prefer not to say
- [ ] Other (please specify): ____________________

50. How many nights did you stay in hospital? *

If more than 10 nights, enter the number below.

____

51. Did you pay out of your own pocket for the hospital stay? *

- [ ] Yes, I paid full
- [ ] Yes, I paid some
- [ ] No
- [ ] I don't know
- [ ] Prefer not to say

66. Please list any more hospital stays below - one admission per line. Do not repeat information you have entered in the previous pages.

	Number of nights (if you did not stay overnight, write "day")	Reason for admission	How much did you pay out of your own pocket? (£/GBP)
1			
2			
3			
4			

	Number of nights (if you did not stay overnight, write "day")	Reason for admission	How much did you pay out of your own pocket? (£/GBP)
5			
6			
7			
8			
9			
10			

Supportive services
This part of the survey is for the person with Parkinson's. Your answers may help to improve services.

67. Over the past 3 months, how many times have you used or received the following services? *

	Never/less than once a month	Once a month	Once a fortnight (two weeks)	Once or twice a week	3-4 times per week	5-7 times per week	Prefer not to say
Nursing assistance at your home	☐	☐	☐	☐	☐	☐	☐
Other home assistance (provided by a paid caregiver, but not a nurse)	☐	☐	☐	☐	☐	☐	☐
Overnight assistance at your home (provided by a paid caregiver, but not a nurse)	☐	☐	☐	☐	☐	☐	☐
Attendance at a day centre	☐	☐	☐	☐	☐	☐	☐
Social event or meal	☐	☐	☐	☐	☐	☐	☐

68. Approximately how much have you or your family spent out of your own pocket on these services (for you) in the past 3 months? (£/GBP) *

	No cost	£1-20/month	£21-£50/month	£51-100/month	£101-£200/month	>£200/month	Prefer not to say
Nursing assistance at your home	☐	☐	☐	☐	☐	☐	☐

	No cost	£1-20/month	£21-£50/month	£51-100/month	£101-£200/month	>£200/month	Prefer not to say
Overnight assistance at your home (provided by a paid caregiver, but not a nurse)	☐	☐	☐	☐	☐	☐	☐
Other home assistance (not provided by a nurse)	☐	☐	☐	☐	☐	☐	☐
Attendance at a day centre	☐	☐	☐	☐	☐	☐	☐
Social event or meal	☐	☐	☐	☐	☐	☐	☐

69. If a paid caregiver comes to your home, how many hours per week did they spend with you, on average, during the last 3 months? Select 0 if paid caregivers did not come to your home. *

	Number of hours per week on average
Nurse	
Other paid caregiver (not a qualified nurse)	

Costs of aids and devices
This part of the survey is for the person with Parkinson's.

70. In the past 3 months, did you or a friend or relative pay for aids or devices out of your/their own pocket? This includes walking frames, grab bars, stair lift, wheel chair, tablet alarms and incontinence products. *

- ☐ Yes
- ☐ No
- ☐ Don't know
- ☐ Prefer not to say

71. How much did the aid or device you/your friends/relatives bought in the last 3 months cost? (£/GBP). This includes walking frames, grab bars, stair lift, wheel chair, tablet alarms and incontinence products.

	Name of aid/device	Cost (£/GBP)
Item 1		
Item 2		
Item 3		
Item 4		
Item 5		
Item 6		

	Name of aid/device	Cost (£/GBP)
Item 7		
Item 8		
Item 9		
Item 10		

Travel expenses
This part of the survey is for the person with Parkinson's.

72. During the past 3 months, have you, your friends or relatives spent any additional money on travel because of Parkinson's? This may include taxis, car park fees, public transport or any other form of travel, for example, in order to attend specialist appointments *

- ☐ Yes, £1-50/month
- ☐ Yes, £51-100/month
- ☐ Yes, £101-200/month
- ☐ Yes, >£200/month
- ☐ No travel costs
- ☐ I don't know
- ☐ Prefer not to say

Other expenses
This part of the survey is for the person with Parkinson's.

73. Have you, your care-partner, family or relatives had any other expenses (not listed in previous questions) that are related to Parkinson's? Leave blank if you have not had any other expenses.

	Item	Cost in £ in the last 3 months
1		
2		
3		
4		
5		

Impact of Parkinson's on employment
This part of the survey is for the person with Parkinson's.

74. Are you currently in paid employment? *

- [ ] Yes
- [ ] No (Including retired)
- [ ] Prefer not to say
- [ ] Other (please specify - e.g. on sick/carer leave):

75. Did you retire from full time work early due to your Parkinson's disease? *

- [ ] Yes, and Parkinson's disease was the main reason that I retired early
- [ ] Yes, and Parkinson's disease was a factor, but was not the main reason I retired early
- [ ] No, Parkinson's disease did not cause me to retire early
- [ ] Prefer not to say

76. How many hours did you work in a typical week, immediately prior to retiring? *

- [ ] Up to 10 hours per week
- [ ] 11-20 hours per week
- [ ] 21-30 hours per week
- [ ] 31-40 hours per week
- [ ] More than 40 hours per week
- [ ] Prefer not to say

77. In a typical week, how many hours do you work? *

- [ ] Up to 10 hours per week
- [ ] 11-20 hours per week
- [ ] 21-30 hours per week
- [ ] 31-40 hours per week
- [ ] More than 40 hours per week
- [ ] Prefer not to say

78. Have you reduced your hours in the last 12 months due to Parkinson's ? *

- [ ] Yes
- [ ] No
- [ ] Prefer not to say

79. How many fewer hours do you work now compared with 12 months ago? *

- [ ] Reduced by <5 hours per week
- [ ] Reduced by 5-10 hours per week
- [ ] Reduced by 11-15 hours per week
- [ ] Reduced by 16-20 hours per week
- [ ] Reduced by more than 20 hours per week
- [ ] I still work the same number of hours per week
- [ ] Prefer not to say

80. In the past 12 months have you reduced the number of hours that you spend on daily activities due to Parkinson's disease? Daily activities include shopping, gardening, cooking and driving, but not paid work. *

- [ ] No, I still spend the same amount of time on daily activities
- [ ] Yes, reduced by <5 hours per week
- [ ] Yes, reduced by 5-10 hours per week
- [ ] Yes, reduced by 11-15 hours per week
- [ ] Yes, reduced by 16-20 hours per week
- [ ] Yes, reduced by more than 20 hours per week
- [ ] Prefer not to say

Impact of Parkinson's on family relationships
This part of the survey is for the person with Parkinson's.

81. Do you feel that Parkinson’s has had an impact on your family relationships? This is in general, taking into consideration all aspects of your relationship. *

- [ ] Not at all

- [ ] Slightly
- [ ] Moderately
- [ ] Very much
- [ ] Extremely
- [ ] Not applicable
- [ ] Don't know
- [ ] Prefer not to say

Any comments?

82. Do you feel that the impact of Parkinson’s on your family relationships has changed as the disease has progressed? This is in general, taking into consideration all aspects of your relationship. *

- [ ] Not at all
- [ ] Slightly
- [ ] Moderately
- [ ] Very much
- [ ] Extremely
- [ ] Don't know
- [ ] Prefer not to say

Additional comments

Other symptoms of Parkinson's
This part of the survey is for the person with Parkinson's.

83. Have you experienced any of the problems below in the past month? Please provide an answer on every line. *

	Yes	No
Dribbling of saliva during the daytime.	☐	☐
Loss or change in your ability to taste or smell.	☐	☐
Difficulty swallowing food or drink or problems with choking.	☐	☐
Vomiting or feelings of sickness (nausea).	☐	☐

	Yes	No
Constipation (less than three bowel movements a week) or having to strain to pass a stool.	☐	☐
Bowel (faecal) incontinence.	☐	☐
Feeling that your bowel emptying is incomplete after having been to the toilet.	☐	☐
A sense of urgency to pass urine makes you rush to the toilet.	☐	☐
Getting up regularly at night to pass urine.	☐	☐
Unexplained pains (not due to known conditions such as arthritis).	☐	☐
Unexplained change in weight (not due to change in diet).	☐	☐
Problems remembering things that have happened recently or forgetting to do things.	☐	☐
Loss of interest in what is happening around you or in doing things.	☐	☐
Seeing or hearing things that you know or are told are not there.	☐	☐
Difficulty concentrating or staying focused.	☐	☐
Feeling sad, 'low' or 'blue'.	☐	☐
Feeling anxious, frightened or panicky.	☐	☐
Feeling less interested in sex or more interested in sex.	☐	☐
Finding it difficult to have sex when you try.	☐	☐
Feeling light-headed, dizzy or weak standing from sitting or lying.	☐	☐
Falling.	☐	☐
Finding it difficult to stay awake during activities such as working, driving or eating.	☐	☐
Difficulty getting to sleep at night or staying asleep at night.	☐	☐
Intense, vivid or frightening dreams.	☐	☐
Talking or moving about in your sleep, as if you are 'acting out' a dream.	☐	☐
Unpleasant sensations in your legs at night or while resting, and a feeling that you need to move.	☐	☐
Swelling of the legs.	☐	☐
Excessive sweating.	☐	☐
Double vision.	☐	☐

	Yes	No
Believing things are happening to you that other people say are not.	☐	☐

Impulse control
This part of the survey is for the person with Parkinson's.

84. Are any of the behaviours listed below an issue for you, or do others think that you have an issue? *

	Yes	No	Prefer not to say
Gambling (casinos, lotteries, internet gambling)	☐	☐	☐
Sex (compulsive urges)	☐	☐	☐
Buying too much or things you don't need	☐	☐	☐
Eating too much	☐	☐	☐
Taking too much of your Parkinson's medication or having trouble cutting down	☐	☐	☐
Spending too much time on hobbies	☐	☐	☐

Sexual relationships
This part of the survey is for the person with Parkinson's.

85. Do you want to answer the next question about your sexual relationships? The person with Parkinson's should answer the next question alone. You may skip this part of the survey if you would prefer not to answer. *

☐ Yes

☐ No

86. In the past 4 weeks, because of your Parkinson's disease, how much of a problem for you were the following issues?

	Not a problem	Little of a problem	Somewhat of a problem	Very much a problem	Not applicable/don't know
Lack of sexual interest	☐	☐	☐	☐	☐
Unable to relax and enjoy sex	☐	☐	☐	☐	☐
Difficult in becoming sexually aroused	☐	☐	☐	☐	☐
Men only: Difficulty getting or keeping an erection	☐	☐	☐	☐	☐
Women only: Difficulty in having an orgasm	☐	☐	☐	☐	☐

Thank you for your participation

87. How did you hear about this survey? *

- [ ] I received an email about it
- [ ] I saw it on social media
- [ ] I saw it on a website
- [ ] I received a flyer from a clinic/health service
- [ ] I received a flyer at an event
- [ ] Other (please specify): ____________________

88. Are you answering the survey alone or is there someone helping you? *

- [ ] I'm alone
- [ ] There is someone helping me (who): ____________________

89. Thank you for your answers. The remaining questions are for your care-partner. If your care-partner is present, please select 'yes'. If your care-partner is only available later, please click 'Save and Continue Later'. If your care-partner does not wish to participate, please select 'no' to exit the survey *

- [ ] Yes, I am a care-partner and would like to answer the care-partner questions
- [ ] No, I would like to end the survey

PART 2: Care-partner survey
This part of the survey is only for the primary care-partner looking after a person with Parkinson's.

90. What is your gender? *

- [ ] Male
- [ ] Female
- [ ] Other
- [ ] Prefer not to say

91. Please select your age range. *

Less than 18
18 – 24
25 – 34
35 – 44
45 – 54
55 – 64
65 – 74
75 – 84
85 and over
Prefer not to say

92. What is your relationship to the person with Parkinson's? *

- [ ] Partner/spouse
- [ ] Parent (mother/father)
- [ ] Sibling (sister/brother)
- [ ] Daughter/Son
- [ ] Friend
- [ ] Prefer not to say
- [ ] Other (please specify): ____________________

93. In the past 3 months, on average, how many hours per week did you spend caring for the person with Parkinson's disease? This may include activities such as doctor appointments, shopping, cleaning, and driving. *

94. Does the person with Parkinson's disease have other family/friends/acquaintances to help them? *

- [ ] Yes
- [ ] No
- [ ] Don't know
- [ ] Prefer not to say

95. In the past 3 months, on average, about how many hours per week does each person (other than you) spend caring for the person with Parkinson's disease? *

	Number of hours per week
Family members	
Friends	
Paid nurse	
Other paid caregiver (not a nurse)	

96. The following questions must be only completed by the care-partner. All information provided will be kept strictly anonymous and confidential. Now, thinking about your experiences caring for a relative/friend with Parkinson's. Please answer never, rarely, sometimes, quite frequently or nearly always for each statement. *

	Never	Rarely	Sometimes	Often	Always/nearly always
Do you feel that your relative asks for more help than he/she needs?	☐	☐	☐	☐	☐
Do you feel that because of the time you spend with your relative that you don't have enough time for yourself?	☐	☐	☐	☐	☐
Do you feel stressed between caring for your relative and trying to meet other responsibilities for your family or work?	☐	☐	☐	☐	☐
Do you feel embarrassed over your relative's behaviour?	☐	☐	☐	☐	☐
Do you feel angry when you are around your relative?	☐	☐	☐	☐	☐
Do you feel that your relative currently affects your relationships with other family members or friends in a negative way?	☐	☐	☐	☐	☐
Are you afraid what the future holds for your relative?	☐	☐	☐	☐	☐
Do you feel your relative is dependent on you?	☐	☐	☐	☐	☐
Do you feel strained when you are around your relative?	☐	☐	☐	☐	☐
Do you feel your health has suffered because of your involvement with your relative?	☐	☐	☐	☐	☐
Do you feel that you don't have as much privacy as you would like because of your relative?	☐	☐	☐	☐	☐
Do you feel that your social life has suffered because you are caring for your relative?	☐	☐	☐	☐	☐
Do you feel uncomfortable about having friends over because of your relative?	☐	☐	☐	☐	☐
Do you feel that your relative seems to expect you to take care of him/her as if you were the only one he/she could depend on?	☐	☐	☐	☐	☐
Do you feel that you don't have enough money to take care of your relative in addition to the rest of your expenses?	☐	☐	☐	☐	☐
Do you feel that you will be unable to take care of your relative much longer?	☐	☐	☐	☐	☐
Do you feel you have lost control of your life since your relative's illness?	☐	☐	☐	☐	☐
Do you wish you could leave the care of your relative to someone else?	☐	☐	☐	☐	☐
Do you feel uncertain about what to do about your relative?	☐	☐	☐	☐	☐
Do you feel you should be doing more for your relative?	☐	☐	☐	☐	☐
Do you feel you could do a better job in caring for your relative?	☐	☐	☐	☐	☐
Overall, how burdened do you feel in caring for your relative?	☐	☐	☐	☐	☐

97. Has your relationship with the person with Parkinson's suffered because of their illness? This is in general, taking into consideration all aspects of your relationship. *

☐ Not at all

- [ ] Slightly
- [ ] Moderately
- [ ] Very much
- [ ] Extremely
- [ ] Not applicable
- [ ] Don't know
- [ ] Prefer not to say

If you would like to provide any details, enter below

98. Do you feel that the impact of Parkinson’s on your relationship has changed as the disease has progressed? This is in general, taking into consideration all aspects of your relationship. *

- [ ] Not at all
- [ ] Slightly
- [ ] Moderately
- [ ] Very much
- [ ] Extremely
- [ ] Not applicable
- [ ] Don't know
- [ ] Prefer not to say

If you would like to provide any details, enter below

99. Please confirm that you are a partner to a person with Parkinson's to ensure you are eligible for the next question which is of a personal nature. *

- [ ] Yes, I am a partner of a person with Parkinson's disease
- [ ] No, I am not a partner of a person with Parkinson's disease

☐ I do not wish to answer questions of a personal nature.

100. Has your sexual relationship with the person with Parkinson's suffered because of their illness? *

☐ Yes

☐ No

☐ Prefer not to say

☐ Not applicable

If you would like to provide any details, enter below

Supplementary Material 2. Patient advocacy groups

Country	Patient advocacy group
France	Fédération Française des Groupements de Parkinsoniens (FFGP)
France	CEPAC
Italy	European Parkinson Therapy Centre
Italy	Accademia LIMPE-DISMOV
Spain	Federación Española de Parkinson
Spain	Hospital Clínico Universitario Santiago de Compostela
Spain	Jefe de Unidad de Enfermedades Neurodegenerativas en Hospital Ramón y Cajal. Madrid
Spain	L'Associació Catalana per al Parkinson (ACAP)
Spain	Párkinson Galicia-Coruña
Spain	Asociación de Parkinson de Ávila
Spain	la Asociación Parkinson Madrid
Spain	Párkinson Ourense
Spain	La Asociación de Parkinson de Villarrobledo
Spain	Department of Neurology, Complejo Hospitalario de Navarra, Pamplona, Spain
Portugal	Associação Portuguesa de Doentes de Parkinson
Portugal	Associação Portuguesa de Doentes de Parkinson (APDPk)
Germany	JuPA
Germany	PARKINSonLINE e.V.
Germany	Parkinson Unna
Germany	Centrum für Neuropsychologische Diagnostik und Intervention (CeNDI)
Germany	HAAG: KLINIKEN KREIS MÜHLDORF A. INN
UK	The Cure Parkinson's Trust
UK	Parkinson's UK
